# The Job Embeddedness Scale: A Test of Its Reliability and Validity in Chinese Perioperative Settings

**DOI:** 10.1155/jonm/9311215

**Published:** 2025-06-29

**Authors:** Tso-kuang Wu, Hung-da Dai, Shu Yu

**Affiliations:** ^1^College of Nursing, National Yang Ming Chiao Tung University, Taipei, Taiwan; ^2^Nursing Department, Taipei Veterans General Hospital, Taipei, Taiwan

**Keywords:** job embeddedness, nurse, retention rate, scale translation, scale validation

## Abstract

**Aims:** This study aimed to translate, validate, and implement the Job Embeddedness Scale (JES) in perioperative settings in Taiwan.

**Background:** Job embeddedness is an important determinant of predicting job turnover; however, no tool exists to measure among perioperative nurses in Taiwan.

**Methods:** A cross-sectional research design was employed using self-administered questionnaires in this study. The JES was forward-translated through group discussions and back-translated by two bilingual translators. Six experts evaluated content validity. A total of 344 perioperative registered nurses from three medical centers were invited to assess the construct validity of the Chinese version of the Job Embeddedness Scale (C-JES), and a confirmatory factor analysis (CFA) was conducted. Reliability analyses were also performed.

**Results:** Based on CFA, our results supported a six-factor version of 31-item C-JES with goodness-of-fit indices that model fit was acceptable. The C-JES presented a high content validity with a CVI of 0.98. Additionally, the 31-item C-JES was highly correlated to the original 40-item JES (Pearson's *r* = 0.98). Cronbach's *α* for the C-JES was 0.91, and test–retest reliability over a 2-week interval showed an intraclass correlation coefficient (ICC) of 0.82.

**Conclusion:** The C-JES is a valid and reliable instrument for assessing job embeddedness among perioperative nurses in Taiwan. This study presents a concise, theoretically grounded 31-item measure that preserves the original scale's full content coverage, demonstrates a strong correlation with the 40-item version (*r* = 0.98), and maintains high internal consistency (*α* = 0.91). Its results can be applied to human resource management and policymaking and then to increase nurses' retention rates. Additionally, this scale can help in understanding different cross-cultural job embeddedness as well as explaining its relationship with nurse retention globally.

## 1. Background

Nurse retention is a significant global challenge. In the United States, the turnover rate for perioperative nurses was 19.9%, with an average of 98 days required to hire a new perioperative registered nurse in 2023. According to the report of the Association of Perioperative Registered Nurses, training an inexperienced nurse for the operating room takes between 6 and 12 months [1, 2]. Replacing a specialty nurse is estimated to cost approximately $64,000 [3], and a new perioperative nurse can add a further US $50,000–75,000 in orientation expenses [1]. This loss, combined with difficulties in recruiting and retaining perioperative nurses, suggests that nurse leaders face a staffing crisis [4].

Traditional turnover studies have focused on attitudinal predictor–job satisfaction, organizational commitment, and perceived ease of movement [5]. A meta-analysis found that these factors explain less than 5% of turnover variance [6], indicating that existing models overlook additional, crucial determinants. Retention strategies should prioritize strengthening nurses' intent to stay rather than reducing dissatisfaction.

Job embeddedness (JE) theory, proposed by Mitchell et al. [7], is defined as a “higher-order aggregate of forces for retention,” encompassing multiple organizational and community factors. JE integrates both on-the-job (e.g., fit, links, sacrifice within the organization) and off-the-job (e.g., community integration and personal ties) influences, offering a more comprehensive view of employee retention [7, 8]. JE is theoretically grounded in sociological concepts of embeddedness [9–11] and Kurt Lewin's field theory [12], which emphasize the influence of social networks, relational constraints, and perceived losses on individual behavior. Holtom and Inderrieden [13] further liken JE to a “web” of interconnected forces that influence whether individuals stay or leave. JE consists of six dimensions across two domains: fit organization/community: perceived alignment with the organization's culture and community environment; link organization/community: formal and informal ties to individuals and groups at work and in the community; and sacrifice organization/community: perceived material or emotional costs associated with leaving the organization or relocating [7, 14, 15]. Research in various nursing settings, including pediatrics, acute care, long-term care, infection control care, and rural and urban environments, consistently shows a negative correlation between higher levels of JE and turnover [16–21]. JE-related factors have been found to explain 13.6%–24.6% of the variance in intention to stay [16, 18] as a predictor of intention to stay [14, 22–25]. JE provides a valuable theoretical foundation for developing targeted, evidence-informed strategies in nursing workforce management. The original Job Embeddedness Scale (JES) consists of 40 items measuring six dimensions: links, fit, and sacrifice across both organizational and community domains. Each item is rated on a five-point Likert scale, with higher scores indicating stronger embeddedness. Previous studies demonstrated the 40 items of JES with a high internal consistency (Cronbach's *α* = 0.87, Table 1) [7, 18]. A seven-item global scale has also been developed [22]. However, the global scale lacks the depth of the original composite scale, particularly in off-the-job factors. In this study, we selected the 40-item JES for validation because it comprehensively captures the multidimensional factors influencing employee retention, offering a broader framework than traditional turnover models. The JES has been translated and validated across various countries and professional groups, teachers in Turkey [26], and banking employees in Pakistan [27]. European countries, Spain, Italy, Denmark, and Finland, shared similar findings in that JE significantly predicted employee turnover [28]. However, no validated Chinese version existed for perioperative nurses in Taiwan—a population facing unique occupational demands and high turnover risk—thereby underscoring the necessity and significance of the present study.

## 2. Methods

**Figure 1:**
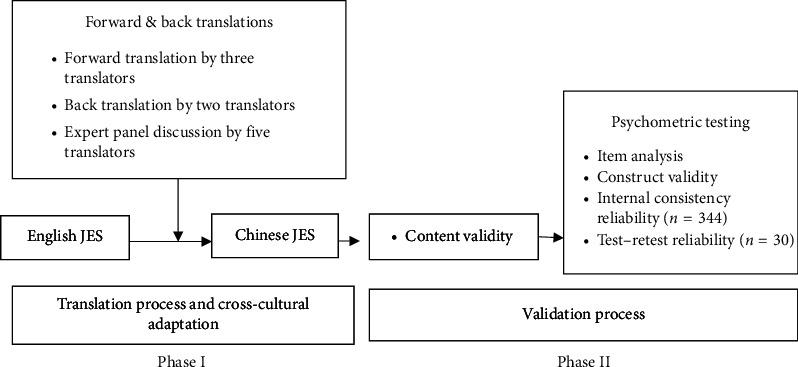
Diagram of translation and validation process of Job Embeddedness Scale.

The original version of the Chinese version of the Job Embeddedness Scale (C-JES) was validated using a two-phase test (Figure 1). Phase 1 analyzed the content validity of the C-JES, whereas Phase 2 tested the scale's construct validity, internal consistency reliability, and test–retest reliability.

### 2.1. Translation and Cross-Cultural Adaptation

#### 2.1.1. Translation Procedure

Prior to translation, formal permission to adapt the JES was obtained from its original author, Professor Mitchell [7]. We adhered to internationally endorsed guidelines for cross-cultural adaptation of psychometric instruments [29]. (1) Three native Chinese translators produced independent drafts: one graduate student familiar with JE theory and two translators neither knew nor understood the concept. Another assistant professor, who was not involved in the forward translation, conducted a comparative analysis of the two translated versions. The translators then met to resolve residual discrepancies and agreed on a Chinese draft. (2) Two Chinese translators, who were fluent in English, independently translated the Chinese version back into English. The two back translators were not familiar with the subject and content of the scale and neither had seen the original English text of the scale. (3) An expert panel—five translators, three nursing scholars, one physician, and one language specialist—reviewed both the forward and back translations to produce the prefinal version.

#### 2.1.2. Cross-Cultural Adaptation

Discrepancies between the back-translated and original English items were discussed and resolved by the translation team to ensure content and conceptual alignment. This rigorous process ensured semantic, content, and conceptual equivalence, resulting in a culturally adapted C-JES suitable for use among perioperative nurses in Taiwan.

### 2.2. Pilot Testing

A pilot study was conducted with 30 perioperative nurses from a northern Taiwan medical center not involved in the main study, using purposive sampling. The researcher distributed sealed, anonymous questionnaires, and participation was voluntary. After 2 weeks, an expert panel reviewed the responses to evaluate item clarity and cultural relevance. Based on their feedback, two items were revised for better cultural alignment: “The perks on this job are outstanding” (organization sacrifice) and “My neighborhood is safe” (community sacrifice). These revisions improved the content validity and cultural suitability of the instrument, resulting in the finalized C-JES.

## 3. Measure

### 3.1. Sample and Setting

Following Tabachnick and Fidell's [30] rule of thumb, at least 300 cases were required for confirmatory factor analysis (CFA). Based on an estimated response rate of 75%, 400 perioperative nurses from three medical centers were invited to participate. The participants included perioperative nurses with at least 1 year of experience, excluding those currently in training, on parental leave, or on long-term sick leave for more than 6 months.

### 3.2. Validation of C-JES

A cross-sectional research design was conducted using self-administered questionnaires in this study. Participants' sociodemographic data were collected, and the C-JES was used to measure JE. The scale uses a five-point Likert scale ranging from 1 (*strongly disagree*) to 5 (*strongly agree*). Following Mitchell et al. [7], a composite score was calculated as the mean of six dimensions, with higher scores indicating higher JE. Mitchell et al. [7] proposed an aggregate measure of embeddedness by computing the mean of six dimensions (a mean of means). Thus, the composite equally weights the influence of the distinct dimensions [7].

### 3.3. Data Collection

Data were collected from March 2023 to August 2023. Participants were informed about the study's voluntary nature and completed self-administered packets, which included the C-JES and a demographic survey. Returning a completed survey in a sealed, prepaid envelope constituted implied consent to use the anonymous data for present and future research. The complete questionnaires were returned in sealed envelopes and sent securely by mail.

### 3.4. Ethical Considerations

The Research Ethics Committee D of National Taiwan University Hospital approved this study (IRB No. 202302026W).

### 3.5. Data Analysis

IBM SPSS Statistics 29 was used for data analysis. Descriptive statistical analysis was conducted for the general participants' data. LISREL 8.8 was used for CFA [31].

### 3.6. Item Analysis

Item analysis was conducted to assess the psychometric adequacy of individual items. An item was considered for deletion if it failed to meet any of the following criteria: (1) the critical ratio (CR) value, with *p* < 0.05, and (2) the item-to-total correlation coefficient lower than 0.30 [32, 33].

### 3.7. Content Validity

To ensure that the C-JES preserved the theoretical integrity of the original instrument, a comprehensive content validation process was conducted. Content validity was assessed through expert judgment. Jury opinion determined content validity. A panel of six experts independently evaluated each item's relevance, clarity, and conceptual equivalence using a four-point Likert scale, scoring each item from 1 (*irrelevant*) to 4 (*absolutely relevant*). Two complementary methods were employed. First, item-level content validity indices (CVIs) were calculated, reflecting the proportion of experts rating each item as “quite relevant” or “highly relevant” [33, 34]. The overall content validity index of the questionnaire (Ave-CVI) was then determined by averaging the CVIs [33]. Additionally, the average scale-level content validity index (Ave-CVI) was computed by averaging all CVI values across items [34]. A CVI above 0.78 for each item and an Ave-CVI below 0.90 were considered indicative of excellent content validity. Second, to further assess the content validity of the C-JES, we adopted the criteria proposed by Colquitt et al. [35], employing the Hinkin and Tracey Correspondence (HTC) method (1999). This approach assesses whether each item clearly represents the intended construct and does not overlap with unrelated constructs. [35]. This rigorous process confirmed that the C-JES items accurately captured the theoretical constructs and were culturally appropriate for use in Chinese healthcare settings.

### 3.8. Construct Validity

To verify the factor structure, a CFA was conducted with a full information maximum likelihood estimation [36] using LISREL 8.8 [31]. Model fit was assessed with comparative fit index (CFI), the non-normed fit index (NNFI) that is also known as the Tucker–Lewis Index (TLI), standardized root mean residual (SRMR) and root mean square error of approximation (RMSEA) [37, 38]. If the CFI > 0.9, NNFI > 0.9, and SRMR and RMSEA ≤ 0.08, then the model fit is considered acceptable [39, 40]. For the comparison between models, the structure with the lowest values of the Akaike information criterion (AIC) and Bayesian information criterion (BIC) was considered the most appropriate [41, 42]. According to Cohen (1988), a Pearson's correlation coefficient (*r*) between 0.50 and 0.70 is considered substantial, *r* ≥ 0.70 is regarded as strong, and values exceeding 0.90 provide extremely strong evidence that two instruments are measuring the same underlying construct [43].

### 3.9. Reliability

Internal consistency was evaluated using Cronbach's alpha, and 30 participants were recruited from a medical center in Taipei. The investigators asked each participant to independently answer the questionnaire on the spot. Cronbach's alpha value was > 0.7 [44], indicating that the reliability of the scale was acceptable. Based on the classification proposed by Oremus et al. [45], the intraclass correlation coefficient (ICC) values below 0.40 are considered poor, between 0.40 and 0.75 fair to good, and above 0.75 excellent [45].

## 4. Results

### 4.1. Sample Characteristics

In Phase 2, 353 perioperative nurses were recruited from three medical centers using purposive sampling to assess validity and internal consistency reliability, representing an 88.3% response rate. We excluded nine participants due to incomplete demographic information and more than five missing items on the scale. The final sample consisted of 344 nurses, with a mean age of 37.9 years (range = 23–64 years, SD = 10.7 years; see Table 2). Most participants were female (92.4%) and held a bachelor's degree (87.2%). On average, they had worked as perioperative nurses for 14.2 years (range = 1–42.4 years, SD = 10.5 years).

### 4.2. Item Analysis

Item analysis was conducted prior to CFA. All inter-item correlations exceeded 0.30, and all corrected the item–total correlations were statistically significant. Critical-ratio *t* tests comparing the upper and lower 27% of respondents were also significant for every item (*p* < 0.05), indicating adequate discrimination. Because no item showed a corrected item–total correlation below 0.30, all items were retained for the subsequent CFA.

### 4.3. Content Validity

A total of six experts participated, four of whom were female, with a mean age of 59.3 years (SD = 9.8) and an average of 34.0 years of professional experience (SD = 6.2). All experts hold either a master's or a PhD degree, with 16.7% having a master's and 83.3% holding a PhD. These experts included a former and current nurse director with expertise in management, a professor familiar with scale development, a former nursing dean, and two nursing professors. Of the 40 items assessed for relevance by the experts, 100% (in = 40) had a CVI > 0.78. The original scale had an Ave-CVI of 98.0%. The HTC value across all items was 0.95, reflecting a high level of expert agreement and supporting the C-JES.

### 4.4. Construct Validity

**Figure 2:**
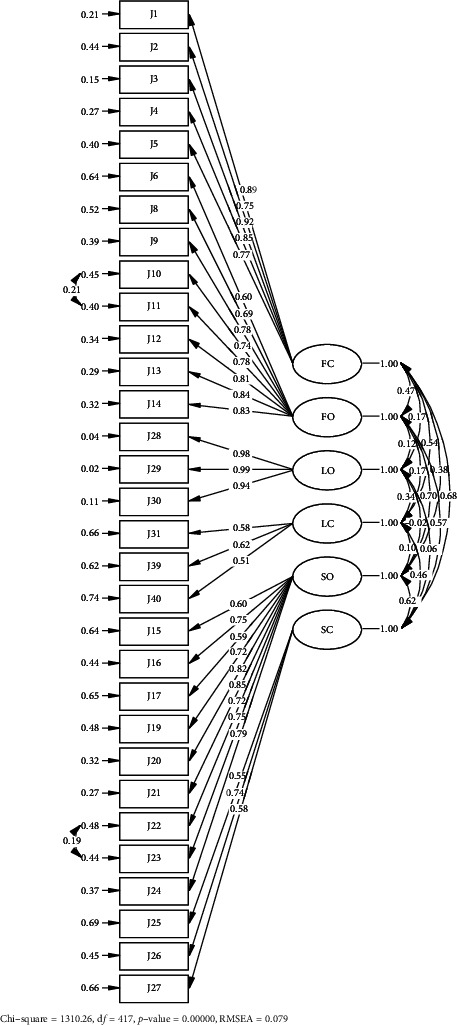
Confirmatory factor analysis for final 31-item Chinese version of the Job Embeddedness Scale. ^∗^FO = fit organization, FC = fit community, LO = link organization, LC = link community, SO = sacrifice organization, SC = sacrifice community.

The initial 40-item C-JES of the CFA revealed that the model fitness was CFI = 0.95, NNFI = 0.91, SRMR = 0.07, RMSEA = 0.09, AIC = 1639.44, and BIC = 1869.97. Nine items with factor loadings below 0.50 were removed, resulting in a final version of 31 items of the C-JES (Table 3). To enhance model fit, the modification indices and item similarity were examined. After accounting for the correlations between the residuals of sacrifice organization items 5 and 6, and fit organization items 7 and 8, the model fit indices improved to CFI = 0.96, NNFI = 0.95, SRMR = 0.07, RMSEA = 0.08, AIC = 1498.94, and BIC = 1763.26 (Table 4, Figure 2). The total scores of the 31-item version and the original 40-item C-JES demonstrated a very strong positive correlation (*r* = 0.98), providing compelling evidence that the abbreviated scale retains the conceptual and psychometric properties of the original instrument. Based on CFA, our results supported a six-factor version of 31-item C-JES.

### 4.5. Reliability

In the present study of RNs working in perioperative nursing care settings, Cronbach's *α* of 0.91 was found for the C-JES. Cronbach's *α* coefficients of the six dimensions were as follows: 0.92 for fit community, 0.92 for fit organization, 0.98 for links organization, 0.56 for links community, 0.91 for sacrifice organization, and 0.64 for sacrifice community (Table 1).

Mitchell et al. [7] found the JE instrument to have an overall Cronbach's alpha of 0.87–0.89. Previous research using a sample of RNs reported a Cronbach's alpha of 0.91 for the JE instrument. Thirty participants completed the C-JES twice, 2 weeks apart. The resulting ICC value of 0.82 indicated an excellent test–retest reliability [45].

## 5. Discussion

The objective of this study was to translate and validate the JES. The JE theory consists of six factors: fit community, fit organization, link community, link organization, sacrifice community, and sacrifice organization. These factors closely align with the dimensions originally proposed by Mitchell et al. [7]. Removing these items highlights unique aspects of Taiwanese perioperative nurses' JE. Perioperative nurses in Taiwan reside outside urban centers due to high housing costs and family obligations. Their heavy workload further weakens their ties with the local community. The demanding and rotational nature of perioperative work also reduces interpersonal continuity and emotional connection to the workplace. As a result, community-based factors may be less salient in shaping JE compared to organizational factors such as teamwork, professional identity, and shared values. Additionally, in collectivist cultures like Taiwan's, the concept of “sacrifice” may be viewed as a shared responsibility rather than an individual loss, potentially affecting the measurement validity of Western-developed items.

In Phase II, instead of using EFA as in an earlier study, this study adopted CFA, which offers methodological advantages for rigorously evaluating a predefined conceptual structure [46], to examine the psychometric properties of the C-JES. By following empirical testing with perioperative nurses, nine items from the original 40-item scale were removed due to low factor loadings below 0.5. The rationale for the exclusion of these items is detailed below.

Fit Organization Factor: One item, “my coworkers are similar to me,” was removed. Link Organization Factor: Four items: “How many coworkers do you interact with regularly?”, “How many coworkers are highly dependent on you?”, “How many teams are you on?”, and “How many committees are you on?” were excluded. Despite frequent interdisciplinary collaboration in perioperative settings, the unpredictable nature of shift rotations and team assignments may dilute the stability of these organizational links [3]. In Taiwan, staffing constraints often require perioperative nurses to rotate across various specialties rather than remain in a fixed unit. Given the complexity of their work and adherence to standardized, task-focused protocols, interpersonal similarities with coworkers may play a less significant role in shaping their sense of JE.

Link Community Factor: Three items, “How many of your close friends live nearby?”, “Are you currently married?”, and “If married, does your spouse work outside the home?”, were removed. Mitchell et al. [7] define links as “formal or informal connections between a person and institutions or other people.” A key tenet of JE is that an increase in the number of links strengthens JE, thereby decreasing turnover [7]. Due to Taiwan's efficient public transportation and the high percentage of unmarried nurses in this study (54.3%), geographic proximity and marital status contribute minimally to community embeddedness. These findings support prior evidence that the number of social ties may not fully explain why people stay in the job [47].

Sacrifice Organization Factor: The item “I would sacrifice a lot if I left this job” was omitted. The perioperative nursing environment is typically characterized by high-pressure, fast-paced, and extended working hours. Nurses with comprehensive perioperative training in medical centers are highly sought after due to their clinical expertise and specialized competencies, leading to a wide range of career opportunities. When dissatisfied with compensation or working conditions, perioperative nurses choose to leave their positions rather than remain, reflecting a high degree of professional mobility within this workforce.

### 5.1. Limitations

The sample consisted exclusively of perioperative nurses recruited from a limited number of medical centers in Taipei, Taiwan, which may restrict the generalizability of the findings to broader nursing populations or other healthcare settings. Future research should include a more diverse sample from different regions, hospital types, and healthcare systems to enhance external validity. Second, the study did not examine criterion validity due to the unavailability of validated Chinese-language instruments measuring theoretically related constructs. This limits the ability to fully establish the scale's construct validity within the Chinese context. Future studies are encouraged to further validate the scale and conduct transcultural comparisons across different geographic regions and countries to explore its cross-cultural applicability.

## 6. Conclusion

JE served as the theoretical foundation for this validation study. A two-phase process was conducted; Phase I involved translation with cultural adaptation; and Phase II comprised validation through content validity, item analysis, CFA, and reliability testing. The 31-item C-JES demonstrated strong internal consistency (*α* = 0.91), had a high correlation with the original 40-item version (*r* = 0.98), and retained full conceptual coverage. Our findings supported a six-factor structure of the 31-item C-JES, established through a robust validation process. The scale is a valid measurement tool and more friendly to use in perioperative settings. It enables nursing administrators to reliably assess JE and its relationship to nurse retention and turnover. Item-level scores can guide targeted interventions, enabling more strategic efforts to enhance JE and improve retention among perioperative nurses.

### 6.1. Implications

The validated 31-item C-JES offers a reliable, culturally appropriate tool for identifying nurses at risk of leaving. Nurse managers can use it to implement targeted, data-driven retention strategies. Finally, this study underscores the importance of cultural adaptation in psychometric assessment. Future research and interventions should consider cultural context to improve measurement accuracy and intervention effectiveness.

## Figures and Tables

**Table 1:** Cronbach's *α* of original 40-item JES and 31-item C-JES.

Subscale	^∗^Original 40-item JES	31-Item C-JES
Fit community	0.79	0.92
Fit organization	0.86	0.92
Link organization	0.62	0.98
Link community	0.50	0.65
Sacrifice organization	0.82	0.91
Sacrifice community	0.59	0.64
Overall scale	0.91	0.91

^∗^Mitchell et al. [7].

**Table 2:** Demographic characteristics of participants (*N* = 344).

Variable	*M*	SD
Age (years; *M* and SD)	37.9	10.7
Number of children (*M* and SD)	0.7	1.0
Nursing experience (years; *M* and SD)	15.2	11.1
OR experience (years; *M* and SD)	14.2	10.5

	***N***	**%**

Gender		
Female	318	92.4
Male	26	7.6
Marital status		
Not married	190	54.3
Married	143	40.9
Widowed	4	1.1
Separated	1	0.3
Divorce	12	3.4
Education		
Vocational school	1	0.3
Associate degree	29	8.4
Bachelor's degree	300	87.2
Master's degree or higher	14	4.1

**Table 3:** CFA of final version factor loading of 31-item C-JES.

Item	Fit community	Fit organization	Link organization	Link community	Sacrifice organization	Sacrifice community
fc1	0.88	—	—	—	—	—
fc2	0.75	—	—	—	—	—
fc3	0.92	—	—	—	—	—
fc4	0.85	—	—	—	—	—
fc5	0.77	—	—	—	—	—
fo1	—	0.60	—	—	—	—
fo2	—	0.67	—	—	—	—
f03	—	0.77	—	—	—	—
fo4	—	0.81	—	—	—	—
fo5	—	0.83	—	—	—	—
fo6	—	0.80	—	—	—	—
fo7	—	0.81	—	—	—	—
fo8	—	0.78	—	—	—	—
lo1	—	—	0.98	—	—	—
lo2	—	—	0.99	—	—	—
lo3	—	—	0.94	—	—	—
lc1	—	—	—	0.58	—	—
lc2	—	—	—	0.62	—	—
lc3	—	—	—	0.51	—	—
so1	—	—	—	—	0.63	—
so2	—	—	—	—	0.72	—
so3	—	—	—	—	0.60	—
so4	—	—	—	—	0.71	—
so5	—	—	—	—	0.75	—
so6	—	—	—	—	0.80	—
so7	—	—	—	—	0.76	—
so8	—	—	—	—	0.79	—
so9	—	—	—	—	0.83	—
sc1	—	—	—	—	—	0.55
sc2	—	—	—	—	—	0.75
sc3	—	—	—	—	—	0.58

**Table 4:** CFA result for 31-item C-JES (*n* = 344).

Index for goodness of fit	Initial value	Final value
*X* ^2^/d*f*	3.55	3.21
RMSEA	0.09	0.08
CFI	0.95	0.96
NNFI	0.94	0.95
SRMR	0.07	0.07
AIC	1639.44	1498.94
BIC	1869.97	1763.26

Abbreviations: AIC = Akaike information criterion, BIC = Bayesian information criterion.

## Data Availability

The data that support the findings of this study are available from the corresponding author upon reasonable request.
